# Sheep recognize familiar and unfamiliar human faces from two-dimensional images

**DOI:** 10.1098/rsos.171228

**Published:** 2017-11-08

**Authors:** Franziska Knolle, Rita P. Goncalves, A. Jennifer Morton

**Affiliations:** Department of Physiology, Development and Neuroscience, University of Cambridge, Downing Street, Cambridge CB2 3DY, UK

**Keywords:** sheep, learning, cognitive testing

## Abstract

One of the most important human social skills is the ability to recognize faces. Humans recognize familiar faces easily, and can learn to identify unfamiliar faces from repeatedly presented images. Sheep are social animals that can recognize other sheep as well as familiar humans. Little is known, however, about their holistic face-processing abilities. In this study, we trained eight sheep (*Ovis aries*) to recognize the faces of four celebrities from photographic portraits displayed on computer screens. After training, the sheep chose the ‘learned-familiar’ faces rather than the unfamiliar faces significantly above chance. We then tested whether the sheep could recognize the four celebrity faces if they were presented in different perspectives. This ability has previously been shown only in humans. Sheep successfully recognized the four celebrity faces from tilted images. Interestingly, there was a drop in performance with the tilted images (from 79.22 ± 7.5% to 66.5 ± 4.1%) of a magnitude similar to that seen when humans perform this task. Finally, we asked whether sheep could recognize a very familiar handler from photographs. Sheep identified the handler in 71.8 ± 2.3% of the trials without pretraining. Together these data show that sheep have advanced face-recognition abilities, comparable with those of humans and non-human primates.

## 1. Introduction

Human face recognition is a critical social skill [1,2]. Humans recognize familiar faces within milliseconds of seeing them [2,3], and can learn to identify unfamiliar faces from repeatedly presented images [4,5]. These skills require both complex image processing and holistic face recognition [6,7]. Sheep are social animals with acknowledged face-recognition skills. They can learn to identify familiar sheep (flock members) from photographs [8–10] and can also recognize known human faces [11]. Little is known, however, about their holistic face-processing abilities, such as whether they can learn to recognize unfamiliar human faces from photographs.

Face-recognition studies in animals have not only provided insights into the cognitive abilities of different species [12,13], but have also been used to probe the neurobiological mechanisms underlying face perception [14,15]. One additional aspect of face recognition is the identification of ‘self’, which has been shown in a variety of species [16]. The ability to recognize same-species faces has been shown in many animals, including chimpanzees [17], rhesus macaques [15,17], cattle [18,19], dairy goats [20,21], pigeons [22], honey bees [23] and sheep [24]. Furthermore, a small number of studies show that some animal species, including rhesus macaques [25], horses [26], dogs [27], mockingbirds [28] and sheep [29] can distinguish faces of individuals from other species (i.e. cross-species paradigm).

Despite the demonstrated face-recognition abilities of some animal species, humans are unquestionably the experts in recognizing faces [30]. One interesting phenomenon is the ability of humans to learn to recognize an unfamiliar face from repeatedly shown images [4,5]. While humans can do this easily, their performance declines significantly when they are asked to identify ‘learned-familiar’ faces when there are changes to specific features of the presentation, for example, the perspective of the presented face [5]. In an early study, Bruce & Young [31] showed that reaction times to select learned-familiar faces were slower when the faces were presented in a new perspective. Choice performance was also less accurate, dropping from approximately 90% correct when tested on the same perspective to approximately 76% correct on a new, tilted perspective. In a related study, it was shown that recognition performance was significantly better when a learned face was presented in the same perspective as the original photograph, rather than in a new, tilted perspective [32]. By contrast, familiar individuals can be identified equally well from photographs irrespective of perspective [33].

Previous research suggests that sheep can learn to discriminate between two-dimensional images of human and sheep faces [11,24,34]. Electrophysiology studies show evidence that neural networks for face perception activated in sheep are similar to those seen in humans and monkeys [35,36]. For example, in a face discrimination study by Peirce *et al*. [11], sheep were trained to discriminate between pairs of photographs of familiar or unfamiliar human faces. Sheep took fewer trials to learn to discriminate between familiar faces than they needed for the unfamiliar faces (27.8 ± 5.7 and 74.6 ± 13.7 trials (±s.e.m.) respectively). From that study, however, it is impossible to conclude that the sheep were actually ‘recognizing’ the individuals. They could simply have learned to discriminate between the two two-dimensional images as shown in other behavioural tasks successfully applied in sheep, such as two-choice discrimination of abstract shapes [37].

In this study, we investigated face-recognition abilities of sheep in a cross-species paradigm, using human faces. Our overarching question was: Are sheep memorizing images, or are they ‘recognizing’ individuals? To answer this question, we first tested their ability to learn to recognize four individuals that they had never seen before (learned-familiar) from repeatedly shown photographs. We then asked if sheep could recognize those faces when they were presented in a new perspective (tilted to the left or to the right). Finally, we asked if sheep were able to recognize a handler with whom they were very familiar (three dimensional) from a photograph (two dimensional). This task requires complex image processing (shift from a three-dimensional representation to a two-dimensional representation) as well as holistic face-recognition abilities [6,7].

## 2. Material and methods

### 2.1. Animals

We used eight sheep for the study (*Ovis aries*, female Welsh Mountain, aged 7–8 years, 45–70 kg). These eight sheep form a single flock of sheep kept at the University of Cambridge. They are held in a separate flock outside all year round, with constant access to grazing and water. They received no food supplements apart from approximately 200 g cereal-based pellets each day (Badminton Country Sheep Nuts, Badminton Country Feeds, UK) during the testing sessions, which usually took place in the morning. The pellets were used as the reward throughout the study. This study was carried out in accordance with the UK Animals (Scientific Procedures) Act 1986 Amendment Regulations 2012, and did not involve any regulated procedures. All eight sheep had previously been used for cognitive testing and were familiar with the semi-automated operant system we used for this task [38,39]; therefore, no habituation phase was necessary. The operant system presents images on computer screens. The sheep had previously seen letters on the screens, but had never been exposed to images of either objects or people.

### 2.2. Operant system and procedure

**Figure 1.**
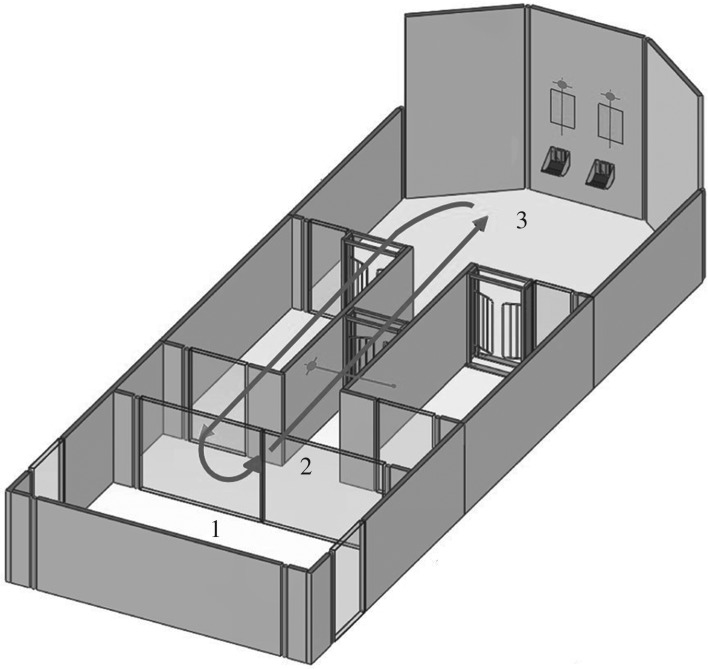
The operant system. All sheep stay in the waiting pen until brought separately in to the pre-testing area (1). They enter the one-way ambulatory circuit (solid arrow) via the entry corridor (2). The sheep self-activate each trial by passing an infra-red sensor in the corridor that leads to the testing area (3). The visual stimuli are presented on two screens in the testing area. An infra-red sensor above each screen captures the selection behaviour of the animal. Activation of the sensor either initiates the dispensing of a food reward into the feed trough or generates an error signal. After receiving a reward (or error signal), the sheep proceeds through a one-way gate to the beginning of the one-way ambulatory circuit. After the session is completed, the sheep is taken back into the pre-testing area (2). From there, the sheep is released into the resting pen. (Adapted from [39].)

The semi-automated operant system in which the sheep were tested allows them to perform the tasks in their own time (electronic supplementary material, video S1). The operant system comprised a maze with a pre-testing and a testing area. From a waiting pen, sheep were brought individually into the pre-testing area (figure 1). From there, the sheep entered a one-way ambulatory circuit via an entry corridor. The ambulatory circuit allowed the sheep to self-pace the task without the interference of an experimenter. The sheep self-activated each trial by passing an infra-red sensor in the starting corridor. From the corridor, the sheep entered the testing area, from where it could see two computer screens with a reward trough underneath each that are integrated into the wall. Visual stimuli were presented on these two screens. An infra-red sensor above each screen and trough captured the choice of the animal. Activation of the sensor either initiated the dispensing of a food reward into the feed trough under the screens for a correct trial or generated an audible signal via speakers above the screens for an incorrect choice or a timeout (i.e. when taking more than 15 s to respond). After receiving a food reward or an error or timeout signal, the sheep proceeded through a one-way gate, back to the beginning of the one-way ambulatory circuit, to initiate the next trial. After the session was completed, the sheep was taken back into the pre-testing area, and was then released into the holding pen.

Sheep were tested in one session of 12 trials (training) per day for 4 days or 15 trials (test-probe) on 1 day. The time taken for each session depended on their pace, and typically lasted 10–20 min. The face-recognition paradigm comprised three training stages and a test-probe. During the training stages, the sheep were taught to discriminate repeatedly presented photographs of four unfamiliar people (learned-familiar faces) from novel unfamiliar people. We used photographs of four celebrities: Emma Watson, former US President Barak Obama, newsreader Fiona Bruce and actor Jake Gyllenhaal. These people were chosen because of the availability of high-quality images in different perspectives via the Internet using the Google-search function.

### 2.3. Paradigm

**Figure 2.**
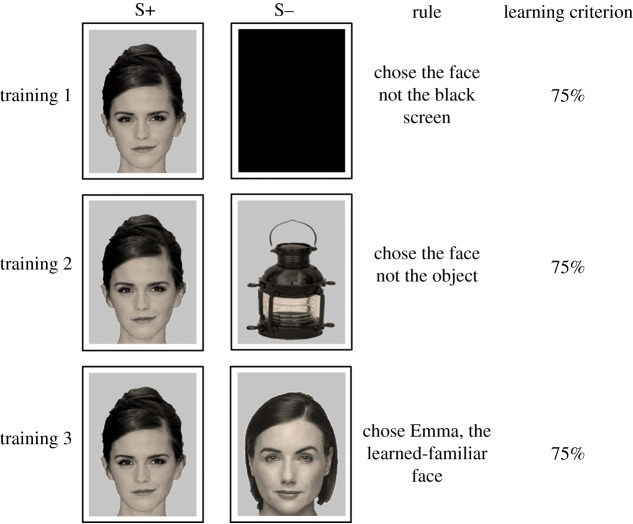
Examples of images that were used for each of the Training stages 1–3. In all stages, a photograph of one of the four celebrities (Emma Watson shown) is the S+. All faces at this stage are shown front-on. In Training 1, the S− is a black screen; in Training 2, S− is an object randomly selected from a set of 62 objects; in Training 3, S− is an unfamiliar face selected randomly from a set of 36 unfamiliar faces. In all Training stages, side (left or right) of presentation of the S+ was pseudorandomized.

**Figure 3.**
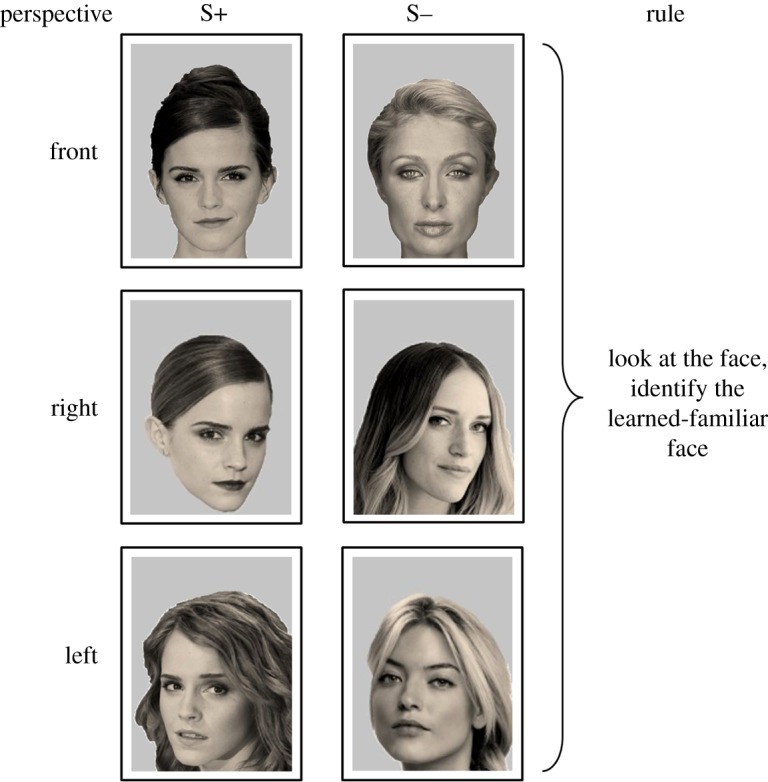
Examples of photographs shown during the test-probe. In the test-probe, a photograph of a learned-familiar face (in this case, Emma Watson shown in the left-hand column) was presented not only face-on, but also in two new perspectives, tilted to the right or to the left side. The learned-familiar faces (S+) were presented against novel unfamiliar faces (S−) in either front-on or tilted perspective. S− images were matched for sex and ethnicity but not hairstyle. The unfamiliar faces were randomly picked from a pool of 48 images different from those used in the training stages. Note that during the task, the images of the four celebrities were each presented an equal number of times, in pseudorandom order, with side (left or right) also pseudorandomized.

During all training stages (figure 2) and the test-probe (figure 3), the sheep were presented with a two-choice task, contrasting a rewarded (S+) and an unrewarded (S−) stimulus. All sheep were exposed to the four different faces during the training and the test-probe. The side of presentation in the system of S+/S− was pseudorandomized. For all training stages, the S+ was a photographic image of one of the four celebrities shown as a front-on face. All sheep were exposed to all four celebrity faces during each session. Celebrity photographs were presented in equal numbers across four sessions, in a pseudorandomized order. In Training 1, the S− was a black screen. In Training 2, the S− was a photograph of an object that was pseudorandomly chosen from a pool of 62 different objects, and in Training 3, it was a novel (unfamiliar) face that was pseudorandomly selected from a pool of 36 images. In all training stages, we included correction trials to prevent side-biases [40]. The images for the S– correction trials were pseudorandomly chosen from two separate pools of images containing 30 new objects or 30 new unfamiliar faces. A correction trial was given on the choice of the incorrect, unrewarded stimulus (S−) or a timeout (where the animal took more than 15 s to make a choice). On the correction trials for Trainings 2 and 3, only the learned-familiar faces (S+) remained the same, whereas the unrewarded stimulus (S−) was chosen randomly from a pool of images of objects (for Training 2) and unfamiliar faces (for Training 3). The S− was different for each correction trial. Objects were items presented as ‘head-sized’ without a background (see the electronic supplementary material, video S2, for example training trials using a lantern, an American football helmet, or an easy chair). Correction trials were repeated until a correct choice was made. On average across all training sessions sheep required 2.7 correction trials with a maximum of 14 (for one sheep) in Trainings 1 and 2. Sheep moved from one training stage to the next when they reached criterion (75% correct in 20 trials or six trials in a row in one session). In each training stage, all sheep completed four sessions of 12 trials plus corrections.

### 2.4. Experiment programming

We used Matlab R2015a (MathWorks, USA) in combination with Psychtoolbox (PTB-3, psychtoolbox. org) to programme all parts of the experiment and capture the behavioural data. Input from sensors and output to feeders and screens were implemented into the Matlab logic, using a 12-bit USB data acquisition device (USB-1208fs, Measurement Computing, USA).

### 2.5. Statistics

We analysed the choice and reaction time data using two-way repeated measure analysis of variance (ANOVA) with Bonferroni's *post hoc* test, unpaired Student's *t*-test, or one-way ANOVA with Newman Keuls *post hoc* test, where applicable. We present mean ± s.e.m. for all data. The threshold for statistical significance was set at *p* ≤ 0.05. All statistical analyses were conducted using SPSS (IBM SPSS Statistics for Windows, v. 22.0, IBM Corp., Armonk, USA), and figures presenting statistical findings were conducted using GraphPad PRISM 5.04 (GraphPad Software, USA).

### 2.6. Stimuli

All images, faces and objects used for this study were drawn from the Internet except for the picture of the very familiar face (i.e. handler) that was taken using a Canon EOS 300 d digital camera. All images were processed using Microsoft PowerPoint 2010 (Microsoft Office Professional Plus 2010, © 2010 Microsoft Corporation) and GIMP 2.8.2 (GNU Image Manipulation Program). All pictures had a resolution of either 96 or 120 dpi, and a depth of 24 bit. All pictures had the same dimensions of 768 × 1024 pixels and were masked to include only the head and hair of the subject. They were recoloured in sepia using a preset recolouring tool of Microsoft PowerPoint, and set on a grey background (RGB (195,195,195)). All images filled 60–65% of the picture space. All facial images were required to have both eyes visible and directed to the camera.

## 3. Results

**Figure 4.**
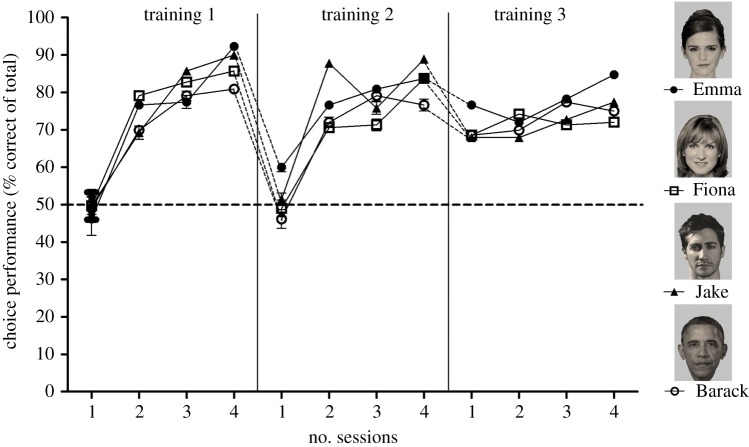
The effect of training on choice performance for selecting one of the four celebrity faces. At all training stages, S+ is one of the celebrity faces shown. S− was a black screen (Training 1) an object (Training 2) or an unfamiliar face (Training 3). Data are shown as the mean ± s.e.m. Where error bars are not visible, they are obscured by the symbols. Chance (50%) is shown as a dashed line.

In Training 1 (figure 2; electronic supplementary material, video S2), sheep were rewarded for choosing the face rather than the black screen. We found no differences between the selection performance on the different faces (d.f. *=* 3, *F* *=* 2.35, *p* = 0.1) but a highly significant improvement across sessions (d.f. *=* 3, *F = *15.47, *p  < * 0.001) in which the sheep improved from 59.0 ± 7.9% in the first to 89.7 ± 4.8% correct in the fourth session (figure 4). These findings are supported by a significant decrease in reaction times between the first and the last session (first session all faces combined: 5.95 ± 0.65 s, last session all faces combined: 3.60 ± 0.44 s; session effect: d.f. *=* 1, *F* = 15.16, *p* = 0.03, face effect: *p* = 0.39, face by session interaction: *p* = 0.99).

In Training 2, sheep were rewarded for choosing one of four celebrity faces, rather than the image of randomly presented objects. The objects were matched in size and brightness; each object was presented only once. There were no differences between the selection performances on any of the individual faces (d.f. = 3, *F* = 0.63, *p* = 0.61), but a significant improvement was seen across sessions (d.f. = 3, *F* = 17.89, *p* < 0.001). Sheep performance improved from 56.6 ± 4.8% in the first to 87.5 ± 4.8% in the last session (figure 4). The reaction times increased from the first to the last session (first session all faces combined: 3.00 ± 0.25 s, last session all faces combined: 4.06 ± 0.25 s; session effect: d.f. = 1, *F* = 14.75, *p* = 0.006, face effect: *p* = 0.08, face by session interaction: *p* = 0.15).

In Training 3, the sheep were rewarded for choosing a learned-familiar face rather than the face of an individual they had never seen before (unfamiliar). The learned-familiar faces were presented the same number of times over four sessions; presentation order of the faces was pseudorandomized. The unfamiliar faces were matched to the celebrity face for sex and ethnicity; each unfamiliar face was presented only once. We found no difference between performance in discriminating the faces of the different celebrities (d.f. = 3, *F* = 1.32, *p* = 0.3) but a significant improvement across sessions (d.f. = 3, *F* = 4.54, *p* = 0.01). Sheep improved from 71.5 ± 1.7% in the first to 79.3 ± 2.7% in the last session (figure 4). In Training 3, the reaction times did not change significantly (first session all faces combined: 3.84 ± 0.48 s, last session all faces combined: 3.67 ± 0.35 s; session effect: *p* = 0.71, face effect: *p* = 0.71, face by session interaction: *p* = 0.074). In fact, they remained similar to the reaction times of the final session in Training 2.

To explore the question of whether reaction times differed between correct and incorrect responses to faces, we generated combined reaction time values across all faces and training stages, with one value for each of the incorrect and correct responses, and the first and last training sessions. Using a 2 × 2 ANOVA (outcome (corr., incorr.) × time (first, last session)), we did not find any significant main effects or interactions. In a Bonferroni corrected *post hoc* test, however, we found that in the first session, response times for incorrect trials were significantly slower than for correct trials (*t* = 2.7, d.f. = 7, *p* = 0.05), which is consistent with the literature [41,42].

Knowing that sheep can learn to recognize unfamiliar faces, we wanted to know whether they would still be able to identify these learned-familiar faces when shown a photographic image of the person taken from a different angle. In the test-probe, the sheep were required to identify the learned-familiar individuals from new photographs in which the faces were present in a new perspective, namely tilted to the left or the right side (figure 3; electronic supplementary material, video S3). The test-probe consisted of one session, presenting three pictures for each celebrity (front, left and right).

**Figure 5.**
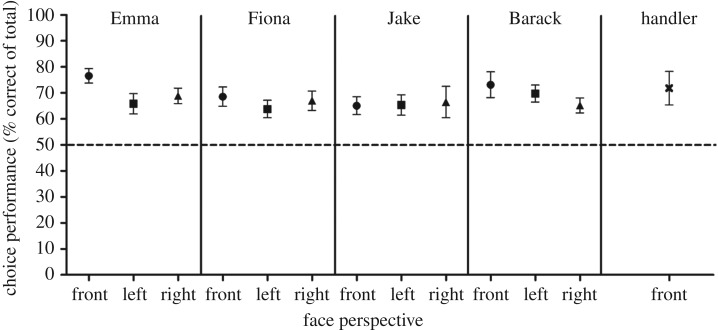
Choice performance for learned-familiar faces of the four celebrities presented in new perspectives, and for the presentation of the face portrait of the handler. Data are shown as mean ± s.e.m. Chance (50%) is shown as dashed line.

In the test-probe, we found that sheep correctly identified the learned-familiar faces, independent of their perspective, 68.0 ± 2.3% of the time (figure 5). This was significantly above chance (d.f. = 7, *t* = 5.39, *p* = 0.004). We did not find any significant differences between the ability of sheep to identify the individual faces of different celebrities (d.f. = 3, *F* = 1.03, *p* = 0.4) or perspectives (d.f. = 2, *F* = 1.79, *p* = 0.2), and there was no interaction between face and perspective (d.f. = 6, *F* = 0.67, *p* = 0.7). For a direct comparison, we created two combined scores, one for the results of the discrimination of the four learned-familiar front-on faces from Training 3, and the other score for all tilted faces (left and right) from the test-probe. Comparing the two scores, we found a significant drop (d.f. = 7, *F* = 5.04, *p* = 0.001) from 79.22 ± 7.5% correct for the front faces to 66.5 ± 4.1% correct for the tilted faces. The sheep's reaction times in response to the new perspectives remained similar to those of the training (front, all faces combined: 3.60 ± 0.39 s; left, all faces combined: 3.36 ± 0.24 s; right, all faces combined: 3.43 ± 0.31 s; face effect: *p* = 0.38, perspective effect: *p* = 0.73, face by perspective interaction: *p* = 0.72).

Having shown that sheep can learn to identify faces of unfamiliar individuals in different perspectives, our final question was whether sheep would also be able to recognize a ‘very familiar’ person from a two-dimensional photographic image. To test this, three copies of a photographic image of one of their regular handlers were randomly interspersed among the stimuli used in the test-probe (four celebrity faces in different perspectives). These were again presented against randomly chosen sex- and race-matched unfamiliar faces in the same perspective. The handler was one of the two principal handlers who trained the sheep daily. She would routinely spend at least 2 h a day with the sheep. The sheep followed the handler voluntarily and fed from her hand. The test-probe contained one session of 15 trials. This included three pictures of the handler, and three pictures of each of the four celebrities in the three perspectives (front, left and right). A different handler ran the experiment. Our results show that the photograph of the handler was chosen over the picture of an unknown person 71.8 ± 2.3% of the time (figure 4). This selection performance was significantly above chance (d.f. = 7, *F* = 9.58, *p* < 0.001) and reaction times were similar to those of the learned-familiar faces (handler: 3.39 ± 0.33 s; face effect: *p* = 0.71).

The reaction of the sheep upon seeing a photographic image of the handler the first time they saw was particularly interesting. Electronic supplementary material, video S4, shows an example of this behaviour. In electronic supplementary material, video S4, the sheep is shown two novel images, one of which is a photograph of a very familiar person, the other is an unfamiliar face. The sheep looks first at the right-hand image, that is, the unfamiliar face. She then moves towards the other face. When the alternative face also turns out to be novel (in that she had never seen an image of this person before), the sheep checks the first (unfamiliar) face again, checks the novel image of the familiar person and then makes a decision to choose the familiar person.

## 4. Discussion

Our training data show that sheep can learn to recognize the faces of unfamiliar individuals from photographs. The test-probe shows that sheep can recognize those individuals even when the image is presented in different perspectives. Finally, the inclusion of the image of the handler's face within the test-probe shows that sheep can identify from a two-dimensional image the face of a very familiar person (handler). Thus, sheep identified both familiar and unfamiliar human faces in a cross-species paradigm with relatively little training (a maximum of 48 trials). Our findings extend the understanding of face-recognition abilities of sheep and suggest that sheep possess holistic face-processing abilities.

Previous work using same-species paradigm [34,35] showed that sheep use information from specific forms of familiarity that were mainly social familiarity (measured as the ability to distinguish between photographs of sheep from the same breed and a different breed). Detailed investigations of unfamiliar face-recognition abilities in animals are rare, even in primates [13]. One study on capuchin monkeys (*Cebus apella*) [43] tested the ability of monkeys to find the ‘odd’ face out of four photographs. In the test-probe of that study, the ‘odd’ face was a familiar (in-group) or an unfamiliar (out-group) face, and the remaining three photographs were either out-group faces (unfamiliar, in different perspectives) or in-group faces (familiar, in different perspectives). The results indicated that monkeys are equally good at identifying the familiar or unfamiliar ‘odd’ face. In this paradigm, Pokorny *et al.* asked whether the monkeys could either detect the one familiar face (in-group) in a group of three unfamiliar faces (out-group), or the one unfamiliar face in a group of three familiar faces. However, as the unfamiliar faces were unrepeated, it was not clear whether or not monkeys would ‘recognize’ the unfamiliar face if it was shown a second time. In that study all faces were presented in different perspectives, although the research question was not aimed at investigating the ability of monkeys to identify the unfamiliar faces presented in different perspectives. Nevertheless, these results indicate that monkeys are able to identify familiar faces independent of their perspective. This has not been investigated previously in sheep. Our study was aimed at answering these open questions.

The results of Training 1 show that sheep can successfully discriminate a photograph of a face from a black screen. This is a simple two-choice discrimination, similar to what has been shown previously [37–39]. Training 2 shows that sheep can recognize a face when presented with a two-choice discrimination between a face and an object. This confirms that sheep can recognize facial features, as shown previously [11]. The reaction times show an increase from the first to the last session, indicating that sheep spent more time looking at images when they are required to discriminate a human face from an object (see, for example, electronic supplementary material, video S2, where the sheep chooses between the portrait of Obama and a football helmet). This would be expected, because the images (object versus face) present a more complex discrimination than face versus blank screen. Training 3 shows that sheep are able to ‘recognize’ the learned-familiar face, which is chosen in preference to that of an unfamiliar face. Although previous studies tested human face recognition in sheep [11,34], in those studies they used extensive training with only a few pictures of familiar people (handler/experimenters). The test-probe used here shows that the learned-familiar face is ‘recognized’ even from a different perspective. This is a novel finding.

In the test-probe, there was a drop in performance of approximately 15% when the front-on faces were changed to tilted faces of the same individuals. This is similar to the reported drop in selection performance seen in humans (from approx. 90% for front-on faces to approx. 76% for tilted faces [4]). Additionally, the ability of sheep to identify the same face in a new perspective was supported by the fact that reaction times in response to the new perspective remained similar to those measured during training when the front-on face image was used. In the test-probe, we also tested whether sheep could recognize a ‘very familiar’ person from a photograph. This task requires the complex image processing of converting three- to two-dimensional information. All sheep chose the image of the handler with performance significantly above chance. This, in combination with the reaction times that are similar to those in response to the learned-familiar faces, support the idea that sheep recognize a familiar human face.

We interpret our data as showing that sheep can not only be trained to recognize unfamiliar human faces, but that they can also recognize the face of a person familiar to them from a two-dimensional image. An alternative explanation, however, is that because the novel stimuli were never rewarded, the sheep are responding negatively to novelty rather than positively to familiarity. While this is possible, we would argue that it is unlikely that the sheep were avoiding novelty. First, in Training 1 sheep have to choose the picture rather than the black screen. They are, therefore, rewarded for picking the image (that is novel) not the blank screen that is more familiar (because the blank screen was the S− for all of the trials in Training 1). Second, in the test-probe, the tilted faces of the celebrities include novel aspects, such as a different hair style or a different facial expression. However, we recognized that in both Trainings 2 and 3, the sheep could, in fact, base their decision on both recognition of familiarity and novelty. Indeed, we think it is likely that this is the case. In the electronic supplementary material, video S4, we show an example of the very first time a sheep sees the novel image of her (familiar) handler. In that video, the sheep appears to be using the information of the unfamiliar face (i.e. ‘I don't recognize this photograph, therefore the other one will be familiar’) to make her decision. She looks first at the unfamiliar face, and moves towards the other face. When the alternative photograph also turns out to be novel, the sheep checks the first (unfamiliar) face again, compares it to the novel image of the familiar person, and then makes her decision to choose the familiar person. It seems likely that while sheep recognize ‘familiar’, they also use ‘unfamiliar’ information to inform their decision-making in a two-choice discrimination task.

Another interpretation of our data in the test-probe is that the high performance is an effect of rapid learning. We do not think, however, that an explanation of rapid learning is sufficient to explain our findings. Each tilted face is only presented once, yet the performance of the sheep over the whole session (12 presentations of the familiar faces in three orientations, plus three presentations of the image of the very familiar face) is significantly above chance. While rapid learning might be an explanation for the high performance in the detection of the very familiar face (the handler) because that was presented three times, recognition of a very familiar face (the handler) seems to be the more likely criterion on which they possibly base their choice decision.

Very few studies have investigated the abilities of animals to recognize handlers or keepers [26,27,44]. Those that have been conducted used species that have extensive contact with humans: namely dogs [27], horses [26] and laboratory chimpanzees [44]. The group of sheep we used here has daily interaction with humans, both during behavioural testing and routine husbandry. This enhanced exposure to human faces could partially explain the excellent performance of the sheep in this task. The extensive human contact is potentially making them human face experts. One could also argue that the general domestication of sheep contributes to their abilities to recognize humans. However, the breed of our sheep, Welsh Mountain, is not one of the more easily managed/tamed breeds. Indeed, they are characterized by their ability to survive independently of human support in the harsh environments in mountain areas. It would be interesting to test this paradigm in a group of sheep that has had comparatively little exposure to human faces.

As well as providing novel ethological insights, this paradigm furthermore provides opportunities for investigating cognitive dysfunction. Indeed, face perception may be impaired at multiple levels in neurodegenerative diseases such as Huntington's disease (HD) [45] and Parkinson's disease [46], as well as psychiatric disorders such as autism spectrum disorder [47] and schizophrenia [48]. The ability to recognize an unfamiliar face, even after it has been presented several times, is impaired in HD patients [45]. Furthermore, HD patients are unable to recognize specific emotional expressions [49], such as disgust, when they are presented without any contextual information [45,50]. Recently, a transgenic sheep model for HD has been developed [51]. These sheep show HD-like brain pathology in the form of aggregates [52], alterations in social behaviours [53] as well as changes in brain and liver metabolism [54,55]. Although it is a well-recognized symptom in HD patients, to date, higher-order behavioural and cognitive processing has not been tested in HD sheep. The face-recognition paradigm presented here would be ideally suited for studying cognitive decline in the transgenic sheep model for HD.

## 5. Conclusion

The results of our study show that sheep have advanced face-recognition abilities, similar to those of humans and non-human primates. Sheep are able to recognize familiar and unfamiliar human faces. Our face-recognition paradigm adds to the existing repertoire of behavioural paradigms suitable for the investigation of cognition and behaviour of farm animals. It would be interesting for future research to include investigation of the abilities of sheep to identify emotional expressions on human faces, possibly in combination with assessing the behaviour in response to the emotional faces, which would provide valuable information for animal welfare. The flexibility of our operant system, which provides height-adjustable screens, make the paradigm we describe suitable for studying cognition in other large animals, such as dogs, pigs, goats and horses. Finally, our face-recognition paradigm provides a means for measuring cognitive function and efficacy of therapeutic agents in sheep models of neurodegenerative diseases such as HD, in which cognitive flexibility is impaired.

## Data accessibility

Data are included as the electronic supplementary material.
